# The American Dental Convention

**Published:** 1865-08

**Authors:** 


### THE AMERICAN DENTAL CONVENTION

Met at White Sulphur Springs, Ohio, on Tuesday, Aug. 1st; and though the number in attendance was not great, a good time was had by those present. The essays read were interesting, and the discussions varied and entertaining. Several Clinics were given; also Microscopic Exhibitions of things very interesting and instructive to those present.

Many suppose that by reading the reports of these meetings, they obtain all that is valuable. A greater mistake could not be made. It is impossible to report a speech or discussion in such a manner as to give the full impression of its first delivery; and then the social personal intercourse is a very important feature of association. Then again, the benefits of demonstrations by clinics illustrations by the microscope, and exhibitions of instruments and appliances, are entirely lost.

Indeed, there is none who can afford to stay away from these conventions. If any one is poor in professional attainments, this is one of the best means of becoming richer; or if he is poor pecuniarily, this is one of the best means of securing confidence and influence; and with these any one can command money. Neither can the man who has the greatest professional attainments, together with an abundance of money, afford to stay away; for even the highest in a profession are far below perfection; and even though they were perfect, they owe a duty to their professional brethren that they cannot in justice disregard; and he who fails in the fulfillment of his duty to another, does injustice to himself.

We have always regarded it as obligatory upon any one in the profession to engage in associative effort for its development. By this means whatever attainments or developments are made become the property of all.

The Convention meets next year in the city of New York, where there will doubtless be a very large meeting.

